# Beyond words: the relevance of autonomy-supportive language in university syllabi

**DOI:** 10.3389/fpsyg.2025.1536821

**Published:** 2025-02-05

**Authors:** Dora Herrera, Aranza Lira-Delcore, Benjamín Lira Luttges

**Affiliations:** ^1^Department of Psychology, Pontifical Catholic University of Peru, Lima, Peru; ^2^Nebrija Research Center in Cognition (CINC), Nebrija University, Madrid, Spain; ^3^Department of Psychology, University of Pennsylvania, Philadelphia, PA, United States

**Keywords:** autonomy-support, control, basic psychological needs, self-determination theory, syllabi, syllabus

## Abstract

**Introduction:**

University syllabi are a semester-long working tool through which professors present a thematic content program, precise assessment mechanisms, and establish the activities schedule, among other tasks. Teachers can promote high-quality motivation among students through syllabi. The goal of this research was to replicate a previous study on the impact of syllabus language on student motivation.

**Methods:**

Two studies were conducted. Study 1 aimed to examine, in 126 first- and second-year students in Humanities General Studies, how they perceive a syllabus with an autonomy-supportive “tone” vs. one with a controlling language. Study 2 explored, in 261 students, basic psychological needs (BPN) satisfaction and their affective approach to the course. Additionally, it investigated the type of motivation (autonomous vs. controlled) that students prioritize when selecting the course.

**Results:**

Findings from Study 1 suggest that autonomy-supportive syllabi are perceived as more attractive, fair, and respectful from the student’s perspective. Results from Study 2 indicate that autonomy-supportive syllabi are related to a better perception of the course, greater BPN satisfaction, and autonomous motivation from students, increasing the probability of them choosing that subject.

**Discussion:**

It is concluded that promoting autonomy, even through the written language shared between teachers and students, is important for improving teaching quality.

## Introduction

Every semester, university students receive academic documents with essential information about the subjects they will study. In these documents, professors present the objectives, content, methods, rules, norms and assessments of the course. Thus, the syllabus serves as a guide to know what to expect from the course and how to achieve the goals set within it (Jones, 2018; Merchán et al., 2022). Therefore, it is an initial communication and introduction between teachers and students, even without having previously met. The syllabus implies for the student a first impression of the teacher (Nusbaum et al., 2021; Young-Jones et al., 2021; Merchán et al., 2022). The “tone” of the syllabus would make a difference in how warm, accessible, and motivated teachers are perceived before starting classes (Jones, 2018; Nusbaum et al., 2021). On the other hand, a syllabus that is poorly organized and has a negative tone might have the opposite effect or be ineffective (Jones, 2018; Merchán et al., 2022).

When students receive a syllabus the *Primacy Effect* (Shail, 2019) may come into play. In general terms, humans have the tendency to register and prioritize information that is presented first. This phenomenon can influence how students perceive the content received when reviewing the syllabus. Consequently, it may reduce uncertainty or either promote or inhibit the appearance of certain behaviors that students prioritize regarding a course and its respective professor (Nusbaum et al., 2021; Merchán et al., 2022).

In the educational context, understanding how the initial information in syllabi influences later student behavior can be further enriched by examining it through the lens of Self-Determination Theory (SDT). According to SDT, behavior arises from motivation, which energizes, directs, and sustains actions toward achieving goals (Reeve, 2018). This psychological process varies in terms of its quality, with individuals experiencing either autonomous or controlled motivation based on their reasons for regulating behavior (Ryan and Deci, 2000, 2017; Pelletier and Rocchi, 2023).

Furthermore, the theory highlights that the quality of motivation is significantly influenced by the satisfaction of three basic psychological needs (BPN): autonomy, competence, and relatedness, to promote personal development. The first one, the need for autonomy, represents individuals’ natural desire to feel volition, freedom of choice when engaging in an activity, and perceived internal locus of causality (Sierens et al., 2009; Van Den Broeck et al., 2010; Kusurkar and Croiset, 2015). The second one, the need for competence, refers to individuals’ desire to feel effective when interacting with their environment (Deci and Vansteenkiste, 2004; Ryan and Deci, 2017). Finally, the need for relatedness refers to individuals’ inherent propensity to interact and connect with others (Deci and Vansteenkiste, 2004; Van Den Broeck et al., 2010). When these needs are met, they foster autonomous motivation, leading to better quality motivation (Deci and Ryan, 2000; Mouratidis et al., 2011). Conversely, frustration of these needs can lead to poorer quality motivation.

It is noteworthy that academic environments play a fundamental role in how the quality of students’ motivation is enhanced. To achieve this, practices can be adopted that satisfy BPN, supporting students’ autonomy. Autonomy support means that teachers facilitate students’ self-established goals and take into account their interests (Sierens et al., 2009; Kaplan, 2018; Reeve, 2018), teach with an interpersonal tone of support and understanding, considering the student’s perspective, creating spaces for opinion, fostering initiative, and teaching in the preferred manner by students (Matos et al., 2018). On the contrary, autonomy can be hindered by adopting a more controlling style (Reeve, 2009). This involves prioritizing the teacher’s perspective over that of the students, using intrusive and oppressive approaches (Reeve, 2009; Vansteenkiste et al., 2012). A controlling style is manifested when the teacher interferes with the student’s thoughts, feelings, or actions, pressuring them to think or behave in a certain way, using controlling language, not tolerating mistakes, and demonstrating authoritarianism (Reeve, 2009; Vansteenkiste et al., 2012).

It should be noted that the previously mentioned phenomenon is neither exclusive to, nor does it only occur during personal interaction within the classroom. The context can either facilitate or hinder the satisfaction or frustration of students’ BPN through the design of syllabi, which, as previously stated, play a fundamental role in students’ expectations of the course and how they approach it and the teacher (Jones, 2018; Nusbaum et al., 2021; Young-Jones et al., 2021; Merchán et al., 2022). Furthermore, the design of a syllabus can help to satisfy these needs, organizing its content in such a way that the future relationship between students and their teachers is favorable and fosters learning based on fundamentally autonomous motivation.

There are various studies that demonstrate the benefits of a syllabus design focused on the student and its impact on students’ perception of the course and their professor (Jones, 2018; Nusbaum et al., 2021). While these studies prioritize visual design elements according to students’ preferences, which can positively influence their academic experience, they do not explore how autonomy-supportive language can have an effect on this experience. To our knowledge, few studies address this aspect within the SDT framework, highlighting motivation and its relationship with the satisfaction or frustration of BPN. Therefore, it is important for teachers to design a more autonomy-supportive syllabus as it can help create a positive first impression of the course and teacher. This language style conveys that the teacher is approachable, understanding and supportive (Jones, 2018; Nusbaum et al., 2021). This positive perception can lead to a stronger student-teacher relationship, which is crucial for effective learning. On the other hand, a syllabus that uses a controlling style could lead to students perceiving the teacher as authoritarian, rigid, and unapproachable. The strategies derived from the controlling style show the opposite approach compared to the autonomous style.

Merchán et al. (2022) conducted an experimental study where university students were divided into two groups: one group was presented with a syllabus that used autonomy-supportive language, while the other received one with controlling language. The results demonstrated that students who were presented with a syllabus using autonomy-supportive language reported significantly higher perceptions regarding the syllabus and the professor in terms of approachability, autonomy, engagement, fairness, and rapport. In contrast, students who received a syllabus using controlling language did not experience similar effects. Furthermore, syllabi adopting an autonomy-supportive approach were seen as more self-directed, adaptable, and provided multiple learning opportunities, among other aspects. These factors also influenced positive perceptions towards the professor, autonomous motivation, and positive feelings towards the course. Similar results were found by Young-Jones et al. (2021) in a sample of university students from the United States. Findings showed that those who received an autonomy-supportive syllabus reported higher levels of autonomy and competence. Additionally, they perceived that the professor would listen to them and take their perspective into account, increasing the likelihood of students enrolling in the course compared to the group with a more controlling syllabus.

Considering the mentioned context and analyzing the learning process of university students, it is crucial to note that autonomous motivation and perceived teacher autonomy support at the beginning of the semester play a significant role in academic engagement (Matos et al., 2018), while controlled motivation increases the likelihood of dropout (Jeno et al., 2018). Merchán et al. (2022) found that students exposed to a controlling syllabus were more likely to withdraw when faced with problems, compared to those who read an autonomy-supportive syllabus. However, to date, no studies have examined the use of autonomy-supportive language in syllabi in Peru.

This is particularly relevant in the context of international research in higher education, as the positive outcomes of BPN satisfaction have been documented across diverse cultural contexts, including both collectivist and non-collectivist societies. These studies, which have investigated all three BPNs—autonomy, competence, and relatedness—show that, even in collectivist countries where perceptions of autonomy may differ, its satisfaction still leads to positive outcomes. This is because, while some define autonomy as a synonym of independence or individualism, SDT understands it as volition, meaning that individuals can autonomously choose to prioritize collectivist practices (Church et al., 2013; Chen et al., 2015). For instance, studies have found evidence of the positive effects of BPN satisfaction in countries such as the United States (Church et al., 2013; Chen et al., 2015), Türkiye (Gülbak and Mutlu Gülbak, 2023), Australia (Church et al., 2013), Spain (Moreno-Murcia et al., 2020), Mexico (Church et al., 2013; Moreno-Murcia et al., 2020), Venezuela (Church et al., 2013), the Philippines (Church et al., 2013), Portugal (Moreno-Murcia et al., 2020), Malaysia (Church et al., 2013), Brazil (Benita et al., 2020; Moreno-Murcia et al., 2020), China (Church et al., 2013; Chen et al., 2015), Israel (Benita et al., 2020), Japan (Church et al., 2013), Chile (Moreno-Murcia et al., 2020), Belgium (Chen et al., 2015), and Peru (Chen et al., 2015; Benita et al., 2020). Therefore, while perceptions of autonomy may vary across cultures, its satisfaction consistently leads to positive outcomes globally, even in collectivist cultures like Peru, showing that it is a universal need. In this context, autonomy-supportive syllabi may also contribute to these positive outcomes, even when considering differences in educational systems, such as between Canada, where the original study was conducted (Merchán et al., 2022), and Peru, where this study takes place. However, the application of SDT in syllabus design remains underexplored.

Therefore, it is relevant to analyze whether addressing students through the syllabus influences their approach to the course and teacher, as well as the role of high-quality motivation, not only in relation to commitment and persistence but also at different moments in the university student’s learning process. This is important because students’ plans and goals often misalign with their motivations (Lens et al., 2012; Herrera, 2019), and dropout rates have increased in Peru (Figallo et al., 2020). For the purposes of this study, the initial interaction between the professor and the student will be analyzed through an academic tool that is constantly used in higher education courses: the syllabus. Specifically, the evaluation that students formulate upon receiving the syllabus as an organized program to develop the course will be recorded.

In sum, this study aims to replicate the quantitative components of the research conducted by Merchán et al. (2022). Some modifications were introduced, including the use of a more contemporary motivation scale that assesses all motivation’s regulations and an effort to obtain a larger sample size. The research is structured into two distinct investigations. Study 1 seeks to examine students’ perceptions of an autonomy-supportive syllabus versus a controlling syllabus. Study 2 aims to explore students’ feelings towards the course, as well as their perception of professors’ need-supportive and need-thwarting behaviors after reading either the autonomy-supportive syllabus or the controlling one. Additionally, Study 2 investigates students’ autonomous and controlled motivation for attending class in each syllabus. The primary focus of the research is on the outcomes of Study 2, with Study 1 serving as a preliminary step to inform the subsequent analysis.

## Study 1

The aim of Study 1 is to confirm whether the syllabi created for the research (adapted from the original study by Merchán et al., 2022) are considered by students as autonomy-supportive or controlling, identifying overall differences between both. Additionally, we sought to understand university students’ perceptions of the syllabus of a hypothetical course using autonomy-supportive language vs. a more controlling language in different dimensions (rapport, engagement, autonomy, approachability, fairness, informativeness, focus and conventionality). It is hypothesized that students will rate the autonomy-supportive syllabus more positively than the controlling syllabus across all dimensions, with the greatest differences expected in the areas of approachability, autonomy, engagement, fairness, and rapport, as identified in Merchán et al. (2022).

### Method

#### Participants

The sample consisted of 126 students from the first 2 years of humanities majors at a private university in Lima, Peru. The study subjects were chosen through non-probabilistic convenience sampling, where access to classrooms was directly used to invite students to participate. Prior to the administration of the questionnaires, informed consent was obtained from the participants.

#### Syllabi design

Two syllabi were developed for a fictitious course titled “Introduction to Human Sciences,” using the syllabus designed for the original study by Merchán et al. (2022) as a model, but the format was adapted to the university where the questionnaires were administered. The two syllabi were identical in terms of thematic content, objectives, summary, and evaluation system. However, variations were established in the language used so that one corresponded to an autonomy-supportive style and the other used a more controlling tone. To ensure that the language used in the syllabi addressed each of the motivating styles, we followed the structure established by Merchán et al. (2022) and referred to the strategies outlined by SDT. These strategies included provision of choice, minimization of pressure, acknowledgment of preferences, provision of rationale and encouragement of decision-making for the autonomy-supportive syllabus, as well as threats, pressure to comply and rigid directives for the controlling syllabus (Ryan and Deci, 2000, 2017; Reeve, 2012) (see Table 1 for examples).

**Table 1:** Examples of language adjustments per syllabus.

Section	AS syllabus	Controlling syllabus
Course format	I have chosen a textbook that I find particularly well-written for our subject. We will cover eleven chapters that students have found most interesting in the past. I encourage you to read each chapter before class (…).	This course will cover eleven chapters from the required textbook, to be reviewed in order throughout the semester. The professor expects to cover at least one chapter per week, and students should complete the readings BEFORE each class.
Laptop use	While a laptop will be a useful tool in our learning environment, it can also be a distraction (…) please use your best judgment when using your laptop.	Students will be asked to leave class if they are using their laptops for activities unrelated to note-taking.
Office hours	I will hold weekly office hours on Tuesdays from 2:30 to 4:30 p.m. and Fridays from 1:00 to 2:00 p.m. (…) My door is open to you if you need it!	By appointment only.
Email policy	Emails will be answered within two business days. I reserve the right to refrain from responding to emails that use disrespectful language.	Emails will only be responded to during weekly office hours. No tutoring will be offered via email.
Absences	I understand that we all occasionally face unexpected situations. In case of absence from the midterm or final exam, please provide me with any legitimate documentation to justify it.	Absences from evaluations without a valid reason will not be tolerated. If you miss an evaluation without explanation, a penalty will be imposed. Reasons such as travel, employment, or errors in reading the exam schedule will not be accepted.

AS, Autonomy-supportive.

Following the approach used in the original study (Merchán et al., 2022), no professor was assigned in the syllabi in order to control for potential external factors. The syllabi were concise and included a summary, course format and objectives, a description of the evaluation system, thematic content, laptop usage rules, teacher’s office hours, email policy, and attendance rules.

#### Procedure

Students were randomly assigned to receive either the autonomy-supportive syllabus (*n* = 69) or the controlling syllabus (*n* = 64). Afterward, participants answered comprehension questions about the syllabus. Those who answered incorrectly to 50% or more of the questions were excluded from the analysis (autonomy-supportive group *n* = 5; controlling group *n* = 2), which led to 64 students for the autonomy-supportive syllabus and 62 for the controlling syllabus. The 50% threshold was adopted to ensure consistency with the methodology used in the original study by Merchán et al. (2022). It allows to confirm that participants had adequately understood the syllabus content, which is critical for accurate evaluations. Subsequently, they rated the corresponding syllabus using the adjective test.

#### Measures

##### Adjective test

The *ad hoc* instrument was constructed for the original study (Merchán et al., 2022), which consists of 17 adjectives with opposite meanings aimed at describing participants’ perceptions after reading the syllabus constructed for the study. The instrument has several subscales that have shown good psychometric properties to measure the following constructs: approachability (*M* = 3.60, *SD* = 1.59), autonomy (*M* = 3.78, *SD* = 1.54, *α* = 0.92), conventionality (*M* = 3.60, *SD* = 1.55), engagement (*M* = 3.39, *SD* = 1.18, α = 0.77), fairness (*M* = 4.73, *SD* = 1.39), focus (*M* = 4.45, SD = 1.66), informativeness (*M* = 5.27, *SD* = 1.64), and rapport (*M* = 4.18, *SD* = 1.58, α = 0.79). For the present study, the instrument was translated and validated. For content validity, it was reviewed by experts, and items were readjusted until an agreement index among judges greater than 80% was achieved. Additionally, reliability analyses were conducted for factors that had more than two items, and it was found that they showed good reliability (autonomy, α = 0.90; engagement, α = 0.71; fairness, α = 0.78; rapport, α = 0.78). Reliability evidence was only evaluated in areas with more than two items.

#### Data analysis

Furthermore, the dimensions were grouped into two broad factors –autonomy-supportive and controlling– to determine the extent to which each syllabus fit into these categories and Student’s independent samples t-test was used to compare both syllabi. The same test was used to compare the means of the dimensions studied in order to evaluate differences in each dimension between the group that received the autonomy-supportive syllabus and the group that received the controlling syllabus.

### Results

Students perceived the autonomy-supportive syllabus more positively, while those who received the controlling language syllabus rated it more negatively (*p* < 0.001; Cohen’s d = 2.14). Specifically, significant differences were found in the perception of approachability, autonomy, engagement, fairness, focus, and rapport.

In terms of approachability, participants who received the autonomy-supportive syllabus perceived it as more relaxed and informal (*M* = 3.71; *SD* = 1.03) than to those who read the controlling language syllabus (*M* = 2.35; *SD* = 0.81). In the dimension of autonomy, where the greatest differences were found (Cohen’s d = 2.14), students who read the autonomy-supportive syllabus perceived it as more flexible, tolerant, providing choice options, and less controlling (*M* = 5.22; *SD* = 1.07) than those who read the control language syllabus (*M* = 2.79; *SD* = 1.22). Regarding engagement, the syllabus supporting autonomy was perceived as more interesting, entertaining, and enjoyable (*M* = 4.60; *SD* = 0.99) than the one using controlling language (*M* = 3.24; *SD* = 0.94). Additionally, participants rated the autonomy-supportive syllabus as fairer and more reliable (*M* = 5.96; *SD* = 0.92) compared to those who read the control syllabus (*M* = 4.70; *SD* = 1.38). Students who received the autonomy-supportive syllabus considered it more student-centered (*M* = 4.58; *SD* = 1.33), while the control syllabus was seen as content-centered (*M* = 3.40; *SD* = 1.80). Finally, regarding rapport, the syllabus using autonomy-supportive language was considered more personal, friendly, and warm (*M* = 5.46; *SD* = 0.97) than the one using more controlling language (*M* = 3.61; *SD* = 1.17). No significant differences were found in the dimensions of conventionality or informativeness (see Table 2).

**Table 2:** Comparison of means between the dimensions of adjectives.

Variable	AS syllabus		Controlling syllabus		*t*	*d*	*df*
	*n*	*M(SD)*	*n*	*M(SD)*			
Approachability	64	3.71 (1.03)	62	2.35 (0.81)	8.24*	1.47	119.26
Autonomy	64	5.22 (1.07)	62	2.79 (1.22)	11.87*	2.12	120.63
Conventionality	63	4.94 (1.57)	61	4.97 (1.51)	−0.11	−0.02	121.98
Engagement	64	4.60 (0.99)	62	3.24 (0.95)	7.89*	1.40	123.99
Fairness	64	5.96 (0.92)	62	4.70 (1.38)	6.00*	1.07	105.59
Focus	64	4.58 (1.33)	62	3.40 (1.80)	4.16*	0.74	112.35
Informativeness	64	6.09 (0.99)	62	5.94 (1.17)	0.82	0.15	119.19
Rapport	64	5.47 (0.97)	62	3.61 (1.17)	9.71*	1.73	118.51

*p < 0.001. AS, Autonomy-supportive.

### Discussion

We found that students perceive the two syllabi differently, in accordance with the experimental manipulation and in agreement with Merchán et al. (2022). Thus, the original study found significant differences between the two syllabi in terms of approachability, autonomy, engagement, fairness, and rapport, while the present study found the same differences along with a significant difference in the perception of focus. Furthermore, the study by Merchán et al. (2022) did not find overall differences in the syllabi when grouping the dimensions into two broad factors –autonomy-supportive and controlling–, which was found in the present research. This provides empirical support to suggest that one syllabus is seen as autonomy-supportive, while the other one is viewed as controlling. This confirms the distinct nature of both syllabi.

Thus, it is demonstrated that the language used in syllabi can have repercussions on students’ perception of the syllabus, being perceived more positively when a language that supports student autonomy is used. This is consistent with Self-Determination Theory, which posits that autonomy-supportive language generates positive outcomes in students compared to a more controlling language (Hsu et al., 2019).

## Study 2

The aim of Study 2 is to investigate student’s feelings about the course and their perceptions of their teacher’s need-supportive behaviors (autonomy, competence, and relatedness support) and need-thwarting behaviors (controlling, competence, and relatedness-thwarting behaviors) following exposure to either a syllabus that uses an autonomy-supportive language or a controlling language. Additionally, it examines students’ overall feelings about the course, specifically focusing on the perceived sense of belonging, perceived relevance of the course for their future, effort invested, persistence, and engagement, in relation to the type of syllabus.

Furthermore, it also examines students’ autonomous and controlled motivation for attending class under each type of syllabus. Therefore, we compared students’ outcomes regarding the syllabus they received.

It is hypothesized that students with the autonomy-supportive syllabus will report higher perceptions of their teachers’ BPN satisfaction compared to the students with the controlling syllabus. Additionally, autonomy-supportive syllabus readers are expected to report more positive feelings about the course compared to the controlling syllabus recipients. Finally, students exposed to an autonomy-supportive syllabus are expected to report higher levels of autonomous motivation for attending class, while those exposed to a controlling syllabus will report higher levels of controlled motivation and amotivation for attending class.

### Method

#### Participants

The sample for this study consisted of 261 undergraduate students in the first 2 years of humanities majors at a private university in Lima, Peru (171 women, 87 men, 3 other). Participants were recruited through the same procedure used in Study 1. Their ages ranged from 18 to 35 years old (*M* = 19.11, *SD* = 2.01). Of these students, 216 attended private schools, while 45 attended public schools. In terms of perceived socioeconomic status, 3 participants identified as belonging to the upper socioeconomic status (SES A, according to the Peruvian system), 60 as upper-middle socioeconomic status (SES B), 156 as middle socioeconomic status (SES C), 34 as lower socioeconomic status (SES D), and 8 as impoverished socioeconomic status or extreme poverty (SES E).

#### Procedure

As in Study 1, Students were randomly assigned to read either the autonomy-supportive syllabus (*n* = 146) or the controlling syllabus (*n* = 136). They were asked comprehension questions about the syllabus afterward. Participants who answered incorrectly to 50% or more of the questions were excluded (autonomy-supportive group *n* = 12; controlling group *n* = 6), resulting in 131 students in the autonomy-supportive group and 130 in the controlling group. Afterwards, they answered the Interpersonal Behaviors Questionnaire, Feelings about the Course questionnaire and the Academic Self-Regulation Scale.

#### Measures

##### Interpersonal behaviors questionnaire (IBQ)

To study the professor’s interpersonal behaviors (Rocchi et al., 2017), a short version of the IBQ was used. This scale is based on SDT basic psychological needs and originally comprises 24 items that measure six factors: autonomy support (AS), autonomy thwarting/controlling (AT), competence support (CS), competence thwarting (CT), relatedness support (RS) and relatedness thwarting (RT). Following the methodology used in the original study (Merchán et al., 2022), one item per factor was chosen to represent behaviors related to constructs associated with each factor: AS: “Give me the freedom to make my own choices in the course”; AT: “Impose their opinions on me”; CS: “Provide valuable feedback”; CT: “Doubt my capacity to succeed in the course”; RS: “Take the time to get to know me”; RT: “Not care about me.” Students had to respond on a scale from 1 to 7 (1 = Completely disagree, 7 = Completely agree) indicating how much each item corresponds to their perception of the teacher after reading the syllabus. Each indicator was analyzed as an individual variable. In this study, the questionnaire underwent translation and validation for the Peruvian context. Experts reviewed it for content validity, and adjustments were made to the items until the judges reached an agreement index exceeding 80%. The need support scale had a Cronbach’s alpha of 0.79, and the need thwarting scale had an alpha of 0.69. Neither would not be improved by the exclusion of any items.

##### Feelings about the course

The *ad hoc* instrument was constructed for the original study (Merchán et al., 2022) to study the perceptions students might have about the course. It consists of six items that measure sense of belongingness (“I feel that I belong in the course”), relevance (“This course is relevant to my future”), self-sacrifice (“I will work hard and postpone recreational activities for the sake of this course”), persistence (“I will not be derailed by setbacks in this course), effort (“I will seek new challenges in learning course material”), and engagement (“I will remain engaged over the whole semester”). The instrument was translated and validated for this study. For content validity, it was reviewed by experts, and items were readjusted until an agreement index among judges greater than 80% was achieved. Reliability analyses were performed for the total scale, showing good internal consistency (*α* = 0.82). Using a Likert scale from 1 to 7 (1 = Completely disagree, 7 = Completely agree), students had to indicate to what extent each of the items corresponds to how they felt about the course after reading the syllabus.

##### Academic self-regulation scale

To measure students’ motivation to attend classes, the revised version of the Academic Self-Regulation Scale (Sierens et al., 2009; Mixan, 2016; Ferreyra, 2017) was used. The original scale, developed by Ryan and Connell (1989, cited in Vansteenkiste et al., 2009), includes a total of 16 items: 4 items for each of the four types of motivational regulation: intrinsic, identified, introjected, and external. These items can also be grouped into: autonomous motivation (intrinsic and identified regulations) and controlled motivation (introjected and external regulations). This scale was adapted to the Peruvian context by Mixan (2016) and further adjusted by Ferreyra (2017), who added 3 additional items to measure academic amotivation. The new items underwent revision by judges and demonstrated an adequate agreement index. Additionally, the scale showed appropriate validity evidence of internal structure (Newton and Shaw, 2013) (KMO = 0.89; χ^2^ = 11285.19) and high reliability (autonomous motivation: α = 0.91; controlled motivation: α = 0.81; amotivation: α = 0.93). The original study (Merchán et al., 2022) employed the Academic Motivation Scale (AMS) (Vallerand et al., 1992) and used one item per subscale (one for each regulation). For the present study, the decision was made to utilize the Academic Self-Regulation Scale due to its widespread use in the SDT field and its strong evidence of psychometric properties, and two items per regulation were chosen. Psychometric properties were analyzed, showing high reliability for autonomous motivation (Cronbach’s alpha = 0.83) and amotivation (Cronbach’s alpha = 0.82) and adequate reliability for controlled motivation (Cronbach’s alpha = 0.58). Using a Likert scale from 1 to 7 (1 = Completely disagree, 7 = Completely agree), students were asked to indicate the extent to which each item corresponded to the reasons why they would attend the syllabus class regularly. They had to answer to statements such as “Because I enjoy doing it” (intrinsic), “Because it is personally important to me” (identified), “Because I would feel guilty if I did not do it” (introjected); “Because I am supposed to do it” (external) and “I do not know, I do not understand why I would attend this course” (amotivation).

#### Data analyses

We used Pearson’s correlation coefficients to examine the relationships between the study variables and demographic characteristics. To address the primary research questions, we employed Welch’s two-sample *t*-tests to compare student perceptions of professors’ interpersonal behaviors, feelings about the course, and academic self-regulation between students randomly assigned to an autonomy-supportive syllabus versus those assigned to a controlling syllabus.

### Results

#### Preliminary results

Initial analyses examined the differences in the participants’ sociodemographic variables (gender, type of school, and perceived socioeconomic status) and found no significant differences, demonstrating the success of the randomization process and the equivalence of both groups.

Subsequent analyses revealed significant associations between the study variables and demographic characteristics. Older students perceived the teacher to be lower in need support (*r* = −0.13*). Male students were less likely to have introjected motivation (*r* = −0.17*), while female students were more likely to exhibit it (*r* = 0.18*); students in middle high social class were less likely to have autonomous motivation for the class (*r* = −0.15*), students in high social class were more likely to have external regulation for the class (*r* = 0.14*). Finally, students in high-middle class were less likely to have identified regulation (*r* = −0.18**), whereas those in poverty were more likely to (*r* = 0.20**). The variable of perceived socioeconomic status was included in the analysis; however, the groups from SES A and SES E were extremely small, preventing a rigorous comparative analysis. Additionally, we conducted multivariate analyses to test whether the effect of the condition varied by demographic factors (see Supplementary Table S1 for details). No significant interaction effects were found, indicating consistency across demographic groups. Consequently, further analyses were performed without accounting for sociodemographic variables as covariates.

#### Professor’s interpersonal behaviors

Group comparison analyses using Welch’s *t*-test indicated a significant difference in perceptions of the teacher’s interpersonal style in supporting BPN, depending on the syllabus they received (autonomy *t* (242.46) = 10.16, *p* < 0.001; competence *t* (234.02) = 7.69, *p* < 0.001; relatedness *t* (241.77) = 8.09, *p* < 0.001) with large effects (Cohen’s *d* = 1.30, 0.98, 1.04). Participants who received the autonomy-supportive syllabus perceived greater autonomy support from the teacher (*M* = 5.04, *SD* = 1.48) compared to those who received the syllabus employing more controlling language (*M* = 3.07, *SD* = 1.54). Similar results were found regarding competence support, with the autonomy-supportive syllabus receiving higher scores (*M* = 5.48, *SD* = 1.17) than the controlling syllabus (*M* = 4.20, *SD* = 1.41). A similar pattern was observed for relatedness support (autonomy-supportive syllabus: *M* = 4.21, *SD* = 1.50; controlling syllabus: *M* = 2.69, *SD* = 1.45).

Regarding the perception of the teacher’s thwarting of BPN, significant differences were found between the two groups (autonomy thwarting/controlling *t* (240.53) = −4.03, *p* < 0.001; competence thwarting *t* (237.10) = −6.24, *p* < 0.001; relatedness thwarting *t* (241.95) = −5.83, *p* < 0.001) with moderate to large effects (Cohen’s *d* = −0.52, −0.80, −0.75). Students who were exposed to the controlling syllabus perceived their teacher as more likely to impose their opinions, thereby thwarting their BPN for autonomy (*M* = 4.00, *SD* = 1.57), compared to those who viewed the autonomy-supportive syllabus (*M* = 3.21, *SD* = 1.49). Similarly, participants with the controlling syllabus perceived their teachers as more likely to doubt their ability to succeed in the course, reflecting greater competence thwarting (*M* = 3.70, *SD* = 1.52) compared to those who received the autonomy-supportive syllabus (*M* = 2.57, *SD* = 1.31). Finally, students with the controlling syllabus perceived that their teacher would be more likely to thwart their BPN for relatedness by showing less concern for them (*M* = 4.03, *SD* = 1.43) compared to those with the autonomy-supportive syllabus (*M* = 2.98, *SD* = 1.41) (see Table 3).

**Table 3:** Comparison of means in professor’s interpersonal behaviors.

Variable		AS syllabus		Controlling syllabus		*t*	*d*	*df*
		*n*	*M(SD)*	*n*	*M(SD)*			
BPN support	Autonomy	131	5.04 (1.48)	130	3.07 (1.54)	10.16***	1.30	246.46
	Competence	131	5.48 (1.17)	130	4.20 (1.41)	7.69***	0.98	234.02
	Relatedness	131	4.21 (1.50)	130	2.69 (1.45)	8.09***	1.04	241.77
BPN thwarting	Autonomy	131	3.21 (1.49)	130	4.00 (1.52)	−4.03***	−0.52	240.53
	Competence	131	2.57 (1.31)	130	3.70 (1.52)	−6.24***	−0.80	237.10
	Relatedness	131	2.98 (1.41)	130	4.03 (1.43)	−5.83***	−0.75	241.95

*p < 0.05, **p < 0.01, ***p < 0.001. AS, Autonomy-supportive; df, degrees of freedom.

#### Feelings about the course

Regarding the differences in students’ perceptions of the course, significant differences were observed between the two syllabi (*t* (217.71) = 5.76, *p* < 0.001; Cohen’s *d* = 0.75). Participants who received the autonomy-supportive syllabus (*M* = 4.81, *SD* = 0.86) reported a greater sense of belonging, perceived relevance of the course for their future, effort invested in the course, persistence, and engagement compared to students who were exposed to the controlling syllabus (*M* = 4.08, *SD* = 1.06).

#### Motivation to attend class

Participants who saw the autonomy-supportive syllabus (*M* = 4.70, *SD* = 1.04) demonstrated higher levels of self-determined reasons (autonomous motivation) for attending classes (e.g., due to enjoyment or perceived importance) compared to those who viewed with the controlling syllabus (*M* = 3.94, *SD* = 1.32). These differences were significant (*t* (206.81) = 4.74, *p* < 0.001; Cohen’s *d* = 0.63). Specifically, students who viewed the autonomy-supportive syllabus exhibited higher levels of intrinsic (*M* = 4.98, *SD* = 1.04) and identified (*M* = 4.42, *SD* = 1.31) regulations of motivation compared to those who received the controlling syllabus (intrinsic regulation: *M* = 4.02, *SD* = 1.46; identified regulation: *M* = 3.86, *SD* = 1.51).

Regarding non-self-determined (controlled) motivation, students who received the autonomy-supportive syllabus (*M* = 3.84, *SD* = 1.07) exhibited similar levels of controlled motivation for attending classes (e.g., due to external pressures or to avoid feelings of guilt) as those who were exposed to the controlling syllabus (*M* = 4.09, *SD* = 1.09). However, despite the lack of statistical significance (*t* (221.38) = −1.57, *p* = 0.119; Cohen’s *d* = −0.21), a pattern emerged suggesting slightly higher scores of controlled motivation among students who viewed the controlling syllabus. Specific analyses of regulatory forms yielded the same results: both groups showed similar results in terms of external (autonomy-supportive syllabus: *M* = 4.03, *SD* = 1.24; controlling syllabus: *M* = 4.30, *SD* = 1.19) or introjected regulation (autonomy-supportive syllabus: *M* = 3.65, *SD* = 1.29; controlling syllabus: *M* = 3.83, *SD* = 1.32).

However, students exposed to the autonomy-supportive syllabus (*M* = 2.37, *SD* = 1.29) exhibited lower levels of amotivation for attending classes compared to those who were exposed to the syllabus with more controlling language (*M* = 2.95, *SD* = 1.51), with this difference being statistically significant (*t* (213.89) = −3.07, *p* = 0.002; Cohen’s *d* = −0.41) (see Table 4).

**Table 4:** Comparison of means in motivation to attend class.

Variable	AS syllabus		Controlling syllabus		*t*	*d*	*df*
	*n*	*M(SD)*	*n*	*M(SD)*			
Autonomous motivation	131	4.70 (1.04)	130	3.94 (1.32)	4.74***	0.63	206.81
Intrinsic reg.	131	4.98 (1.04)	130	4.02 (1.46)	5.62***	0.75	197.15
Identified reg.	131	4.42 (1.31)	130	3.86 (1.51)	2.93**	0.39	215.04
Controlled motivation	131	3.84 (1.07)	130	4.09 (1.09)	−1.57	−0.21	221.38
Introjected reg.	131	3.65 (1.29)	130	3.83 (1.32)	−1.02	−0.14	221.29
External reg.	131	4.03 (1.24)	130	4.30 (1.19)	−1.69	−0.23	222.00
Amotivation	131	2.37 (1.29)	130	2.95 (1.51)	−3.07**	−0.41	213.89

*p < 0.05, **p < 0.01, ***p < 0.001. AS, Autonomy-supportive; reg., regulation; df, degrees of freedom.

### Discussion

These study findings largely support the initial hypothesis that students that receive an autonomy supportive syllabus would perceive their professor more positively, feel better towards the course and report higher levels of autonomous motivation. These results are aligned with the SDT framework, which suggests that the environments that support the BPN foster better motivational and affective outcomes (Ryan and Deci, 2011; Ryan, 2023).

In this sense, the hypothesis that an autonomy-supportive syllabus would lead to higher perceptions of BPN support—autonomy, competence, and relatedness—was strongly confirmed by the data. Students exposed to autonomy-supportive syllabus reported higher levels of need support, and those who viewed the controlling syllabus reported more levels of need thwarting. These results are consistent with findings from Merchán et al. (2022) and Young-Jones et al. (2021) in syllabi design, and many other research in SDT (e.g., Reeve, 2009; Vansteenkiste et al., 2012). The large effect sizes observed further reinforce the robustness of these outcomes.

These findings underscore the importance of the tone used in syllabi on how students anticipate their interaction with their professor and even with the course. Thus, the results also show that the autonomy-supportive syllabus improved the perceptions of the students towards the course, leading them to feel a greater sense of belonging, perceived relevance, and engagement. These findings align with those by Matos et al. (2018), who highlighted the importance of autonomy support in academic engagement.

Moreover, the study also demonstrates the relevance of an autonomy supportive-language as a predictor of better-quality motivation, specifically self-determined (autonomous) motivation, which has been linked to better academic outcomes (Ryan and Deci, 2017). In contrast, the syllabus that used a controlling tone did not affect the different regulations of controlled motivation as it was hypothesized, but it did result in greater levels of amotivation compared to the autonomy-supportive syllabus. This reflects the adverse effects of controlling language, which, according to SDT, can undermine students’ intrinsic motivation and overall engagement. This is consistent with Reeve’s (2009) findings, which show that controlling environments can lead to the loss of interest, resistance and withdrawal from imposed tasks, which can make them less engaged and diminish their motivation.

The findings provide a series of theoretical and practical implications and contributions. Results contribute to the growing literature on SDT, specifically in syllabus design, which is a relatively unexplored area. Additionally, it offers practical recommendations for syllabus design, such as using phrases that invite students to explore topics in a way that aligns with their interests (supporting autonomy), offering clear rationales and objectives for grading criteria (enhancing competence), and emphasizing accessibility for teacher support (fostering relatedness), among other strategies, such as the ones detailed in Table 1. Beyond these practical suggestions, these findings also allow us to understand how the first impression created by a syllabus can set the tone for the entire semester, influencing not only how students perceive their instructors but also their future engagement and dedication in the course, as well as their future choice of professors, in an empirical manner.

Despite the contributions, this study presents some limitations. The information was gathered with self-report questionnaires, which can always present certain difficulties, such as social desirability, response biases, and do not record real behaviors (e.g., class attendance or academic performance). However, self-report data are not only cost-effective and efficient, but also provides access to personal insights which allows us to deeply understand students’ perceptions, which was the end-goal of this study. On the other hand, the study employed a purposive, non-probability sampling, representing a very specific university population, which may limit the generalizability to other educational contexts. Even though this approach is common in exploratory research, it limits the application of the findings to a more diverse sample. Therefore, future research could benefit from a broader, more random sample which could improve the external validity of the results. Additionally, this study focuses on the Peruvian context, which could limit the transferability of the findings. However, research has consistently shown that BPN satisfaction is universal and beneficial across diverse cultural settings (Church et al., 2013; Chen et al., 2015; Benita et al., 2020; Moreno-Murcia et al., 2020; Gülbak and Mutlu Gülbak, 2023). The applications of these findings to syllabus design, though, remains unexplored, therefore, future research should study how BPN satisfaction can be applied in syllabi design across different cultural contexts. Finally, while it provides evidence of the state of the relationship between the variables, further research could delve further regarding participants’ experiences with qualitative, longitudinal and experimental research, providing a deeper understanding of how students’ perceptions change over time and provide insights into the long-term impact of syllabus design on engagement and academic outcomes.

To summarize, the findings of this study highlight the crucial importance of a syllabus design that supports students’ autonomy, not only in its impact on their perception of how the course could be in the future and their professor, but also in their motivation to attend the class if enrolled in the course. These results reinforce the idea that the use of an autonomy-supportive language and tone in educational materials can undertake a crucial role in the creation of more positive and effective learning environments, as well as students’ engagement (Matos et al., 2018). This study offers valuable insights in the international research field of higher education by demonstrating how SDT can be applied in the language used in documents across different cultural and educational settings. The findings from the original study by Merchán et al. (2022) in Canada, along with the present study in Peru, expand the understanding that autonomy support is universally important for student motivation and learning outcomes. Therefore, this research contributes to SDT as it highlights how small and precise interventions, such as the tone used in an academic document, can have significant effects on how the students’ approach their learning process. This may not only improve their academic performance, but also their motivation quality which can have positive long-term effects in their academic experience.

## Data Availability

The raw data supporting the conclusions of this article will be made available by the authors, without undue reservation.
