# Supplementary material for: Beyond words: the relevance of autonomy-supportive language in university syllabi
Source: Front Psychol. 2025 Feb 5;16:1536821. doi: 10.3389/fpsyg.2025.1536821 (PMC11870025; doi:10.3389/fpsyg.2025.1536821)
Supplement: Supplementary file 1 [file Table_1.docx]

**SUPPLEMENTARY MATERIAL**

**Supplementary Table S1**. Interaction Effects by Moderator: Results of the Multivariate Analysis

| **Moderator** | **Interaction Term** | ***b*** | **SE** | ***t*** | ***p*** |
| --- | --- | --- | --- | --- | --- |
| BPN Support | Condition * Sex–Woman | .291 | .308 | .946 | .922 |
|  | Condition * Sex–Other/rather not say | .620 | 1.398 | .444 | .930 |
|  | Condition * Age | .061 | .073 | .828 | .922 |
|  | Condition * Public school | -.064 | .388 | -.166 | .954 |
|  | Condition * Lower SES | .466 | .465 | 1.003 | .922 |
|  | Condition * Middle SES | .589 | .349 | 1.691 | .922 |
| BPN Thwarting | Condition * Sex–Woman | -.372 | .303 | -1.228 | .922 |
|  | Condition * Sex–Other/rather not say | -.250 | 1.374 | -.182 | .954 |
|  | Condition * Age | -.041 | .073 | -.566 | .922 |
|  | Condition * Public school | .010 | .383 | .027 | .979 |
|  | Condition * Lower SES | -.507 | .461 | -1.098 | .922 |
|  | Condition * Middle SES | -.091 | .346 | -.263 | .954 |
| Feelings About the Course | Condition * Sex–Woman | .369 | .269 | 1.373 | .922 |
|  | Condition * Sex–Other/rather not say | -.215 | 1.195 | -.180 | .954 |
|  | Condition * Age | .046 | .064 | .723 | .922 |
|  | Condition * Public school | .290 | .337 | .861 | .922 |
|  | Condition * Lower SES | .194 | .406 | .478 | .930 |
|  | Condition * Middle SES | -.127 | .309 | -.410 | .930 |
| Autonomous Motivation | Condition * Sex–Woman | .246 | .337 | .729 | .922 |
|  | Condition * Sex–Other/rather not say | .813 | 1.483 | .548 | .922 |
|  | Condition * Age | .022 | .086 | .251 | .954 |
|  | Condition * Public school | .042 | .430 | .098 | .954 |
|  | Condition * Lower SES | -.307 | .508 | -.603 | .922 |
|  | Condition * Middle SES | -.364 | .381 | -.955 | .922 |
| Controlled Motivation | Condition * Sex–Woman | .179 | .305 | .586 | .922 |
|  | Condition * Sex–Other/rather not say | -1.607 | 1.342 | -1.197 | .922 |
|  | Condition * Age | -.113 | .078 | -1.448 | .922 |
|  | Condition * Public school | -.052 | .392 | -.132 | .954 |
|  | Condition * Lower SES | -.860 | .468 | -1.839 | .922 |
|  | Condition * Middle SES | -.504 | .350 | -1.438 | .922 |

*Note:* The tests adjusted the false discovery rate using the Benjamini-Hochberg correction. No interaction effect was significant.
